# Application of Cloud Simulation Techniques for Robotic Software Validation

**DOI:** 10.3390/s25061693

**Published:** 2025-03-09

**Authors:** Diogo Vieira, Miguel Oliveira, Rafael Arrais, Pedro Melo

**Affiliations:** 1 Department of Mechanical Engineering, University of Aveiro, 3810-193 Aveiro, Portugal; mriem@ua.pt; 2 Institute of Electronics and Informatics Engineering of Aveiro (IEETA), University of Aveiro, 3810-193 Aveiro, Portugal; 3 Intelligent Systems Associate Laboratory (LASI), University of Minho School of Engineering, 4800-058 Guimarães, Portugal; 4 Institute for Systems and Computer Engineering, Technology and Science (INESC TEC), 4200-465 Porto, Portugal; rafael.l.arrais@inesctec.pt (R.A.); pedro.m.melo@inesctec.pt (P.M.); 5 Faculty of Engineering, University of Porto, Rua Dr. Roberto Frias, 4200-465 Porto, Portugal

**Keywords:** cloud robotics, ROS, AWS, RoboMaker, continuous integration, continuous deployment

## Abstract

Continuous Integration and Continuous Deployment are known methodologies for software development that increase the overall quality of the development process. Several robotic software repositories make use of CI/CD tools as an aid to development. However, very few CI pipelines take advantage of using cloud computing to run simulations. Here, a CI pipeline is proposed that takes advantage of such features, applied to the development of ATOM, a ROS-based application capable of carrying out the calibration of generalized robotic systems. The proposed pipeline uses GitHub Actions as a CI/CD engine, AWS RoboMaker as a service for running simulations on the cloud and Rigel as a tool to both containerize ATOM and execute the tests. In addition, a static analysis and unit testing component is implemented with the use of Codacy. The creation of the pipeline was successful, and it was concluded that it constitutes a valuable tool for the development of ATOM and a blueprint for the creation of similar pipelines for other robotic systems.

## 1. Introduction

In the field of robotics, the use of cloud computing and cloud resources is becoming more common, as of late. The use of these cloud-based tools brings several benefits to the development of robotic software. For example, by using cloud simulation services, several simulation jobs can be run simultaneously. Additionally, the overall local processing power needed decreases, due to the use of remote resources. With this in mind, Amazon Web Service (AWS) has developed a service for the issuing of simulation jobs on the cloud, called RoboMaker (https://docs.aws.amazon.com/robomaker/latest/dg/chapter-welcome.html (accessed on 4 March 2025)).

Continuous Integration (CI) is a methodology for software development where updates are frequent and automatically tested. This work is inspired by the idea of establishing a methodology for the automatic testing and validation of robotic software, by means of a CI pipeline that makes use of cloud simulations for testing. GitHub Actions (https://docs.github.com/en/actions (accessed on 4 March 2025)) is a tool that allows for the creation of these pipelines. A process called Continuous Deployment (CD) can serve as an addendum to a CI pipeline. This process sees the developed software be automatically deployed to the consumer after it is tested by the CI process.

The Robot Operating System (ROS) (https://www.ros.org/ (accessed on 4 March 2025)) is the de facto standard framework for the development of robotic software. It consists of an open-source set of tools for development, and allows the use of multiple programming languages, such as Python and C++ [1].

A robotic application developed at the University of Aveiro is the Atomic Transformation Optimization Model (ATOM) (https://lardemua.github.io/atom_documentation/ (accessed on 4 March 2025)). This is a calibration tool designed to calibrate generalized robotic systems [2]. This application will be used as a testing target to validate our proposed approach.

Multiple ROS repositories use CI/CD techniques. However, only one approach to the use of such tools was found that made use of cloud simulations using AWS RoboMaker, proposed by Teixeira et al. [3].

Adding further context to this paper, a tool that facilitates the containerization of ROS workspaces has been developed, called Rigel (https://github.com/rigel-ros/rigel (accessed on 4 March 2025)) [4]. Rigel also simplifies the orchestration of containers for the testing of robotic software in said containers.

Proposed in this paper is a new CI pipeline that integrates cloud simulations using AWS RoboMaker, a static code analysis and unit testing component, and uses Rigel to facilitate the testing and issuing of cloud simulations on RoboMaker. The target of this pipeline’s testing is ATOM. This pipeline was implemented in a publicly available repository, found here (https://github.com/Kazadhum/atom (accessed on 4 March 2025)).

The usage of Rigel in the creation of such pipelines, along with the integration of cloud simulations in the testing pipeline, should make this pipeline a powerful tool for ATOM’s developers. In addition, though the proposed pipeline is specific to ATOM, it would also be a blueprint for similar pipelines for the testing of different robotic applications.

Using the proposed CI pipeline benefits the research work on calibration as it allows several researchers (at the time of writing, ATOM has around 15 contributors) to work in parallel on their research lines, while maintaining the core base of the calibration framework bug free due to the periodical tests that are carried out automatically. Having the calibration of a robotic system be systematically tested as part of a series of verifications has the potential to make the ATOM framework more stable and easily maintained.

### State of the Art

The benefits of using cloud resources in robotics were studied by Guizzo [5] in 2011. Chief among these is the fact that, by using cloud resources, fewer computational resources are needed and, as such, lighter robotic systems can be used. One possible application for these resources lies in, for example, the training of neural networks. The significant computational cost required to train certain networks makes the use of cloud resources appealing [6].

When it comes to the perks brought by the introduction of CI/CD pipelines in software development, there are several. Among these are better communication between developers and clients, since feedback is faster due to the CD process, and a general improvement in the quality of the development, due to automatic testing. Furthermore, the CI process helps in the detection of errors early in development [7,8]. Tools exist that facilitate the application of CI/CD in ROS applications, such as the ROS Build Farm (http://wiki.ros.org/buildfarm (accessed on 4 March 2025)) and ROS Industrial CI (https://github.com/ros-industrial/industrial_ci (accessed on 4 March 2025)) [9].

A framework that includes CI processes for the testing of racing software was presented by Jiang et al. [9]. However, even though AWSs are used in this pipeline and the racing software, named AutOps, is containerized, RoboMaker is not used and no simulations are issued on the cloud. Containerization is a necessary step to using RoboMaker and will be explained further in this paper.

As mentioned earlier, Teixeira et al. [3] proposed a solution for the integration of RoboMaker simulations in a CI/CD pipeline. The process is triggered after a change is made to the source code. Afterwards, ROS Industrial CI is used to run unit and integration tests. Following that, a bundle is created, called *robot application*. Generally, the *robot application* includes everything that is supposed to be tested by the cloud simulations. This bundle is paired with another, called *simulation application*, and both are uploaded to the Amazon Web Service Short Storage Service (AWS S3) to then be used by RoboMaker. It should be noted that, at the time of writing, RoboMaker does not use bundles stored in AWS S3 anymore but does use container images stored in the Amazon Web Service Elastic Container Registry (AWS ECR). After the cloud simulations are finished, the results are analyzed. If the simulation proves successful, the CD process is triggered and a container image is deployed.

Containers have been mentioned several times as they are integral parts of this process and are, in fact, currently needed to issue simulations in RoboMaker. A container is a unit of software that combines code and dependencies. The use of containers in software development brings forth several boons. For example, since a container includes a software’s dependencies, the software can be run regardless of the system it is run on [10]. Furthermore, this can ease the sharing of code and lead to increased modularity [11]. One of the typical tools used to containerize software is Docker.

A container is generated by running a container image and an image is generated based on what is known as a *Dockerfile*. When it comes to orchestrating or launching multiple containers, a YAML file is required as input to a tool named Docker Compose (https://docs.docker.com/compose/ (accessed on 4 March 2025)). A pipeline was proposed by Melo et al. [12] that generates these YAML files automatically. Furthermore, this pipeline also creates the Dockerfiles necessary to containerize ROS workspaces based on another YAML file that is given as input to the pipeline.

In their later work, Melo et al. [4] proposed an improved solution: Rigel. Rigel can create container images from entire ROS workspaces and orchestrate several containers, in order to test the robotic software. Rigel can also perform the deployment of container images to remote container registries, like Docker Hub (https://hub.docker.com/ (accessed on 4 March 2025)) or AWS ECR. Rigel is a plugin-based tool, where each plugin performs a specific task and it is configured via what is known as a *Rigelfile*.

Containerization is present in a robotic software development paradigm proposed by Lumpp et al. [11]. In this methodology, the software is containerized and then simulations are run on the robot’s hardware. Hence, though the benefits of containerization are taken advantage of, the same cannot be said for cloud simulations.

One of the components to be integrated in our proposed approach is static code analysis. This is the process of verifying that code is compliant with certain rules or guidelines without running it [13]. This process helps in the detection of problems in the earlier development stages [13,14]. Tools like Codacy (https://www.codacy.com/ (accessed on 4 March 2025)) exist that perform static analysis on code repositories. Furthermore, Santos et al. [15] have created a tool that performs static code analysis, intended to be used in ROS workspaces, named the High Assurance ROS Framework (HAROS) (https://github.com/git-afsantos/haros (accessed on 4 March 2025)). While most static analysis tools only analyze individual ROS nodes, HAROS takes into account complete ROS applications.

Yet another component to be included in this proposed pipeline is unit testing. A *unit test* tests a single unit of code [16,17]. Niedermayr et al. [16] differentiate these tests from *system tests*, which test the entire system at once instead of individual code units. Typically, this means running a unit of code, such as a method or function, and comparing its output with what is to be expected. A tool for running unit tests for Python source code, which will be used in our pipeline, is the built-in unittest (https://docs.python.org/3/library/unittest.html (accessed on 4 March 2025)) module. *Code coverage* is a way of measuring how covered a module of code is by unit tests [18]. Several metrics may be used for code coverage. One of these is *line coverage*, or the ratio of lines covered [18]; an alternative is the ratio of functions or methods that are run when running the unit tests [16]. Codacy can also display the results of a code coverage report, uploaded as part of a CI pipeline.

The worth of unit testing and code coverage reports was studied by Niedermayr et al. [16]. They concluded that code coverage, using method coverage as a metric, is indeed a useful metric when measuring the extent of unit tests and not system tests. In the case of system tests, the authors warn of the possibility of pseudo-tested methods. This occurs when, for example, a test is poorly written so that it does not effectively test a function but still counts towards coverage. The authors state that the occurrence of these pseudo-tested methods is higher in the case of system tests than in the case of unit tests.

A study was conducted at Google in 2019 by Ivankovi et al. [18], with the purpose of understanding how useful the code coverage tools were considered by the developers. They conclude that these tools are indeed used by most developers when readily available. The authors also give recommendations for their integration in development, these being that the tools should operate automatically and as effortlessly as possible.

**Figure 1:**
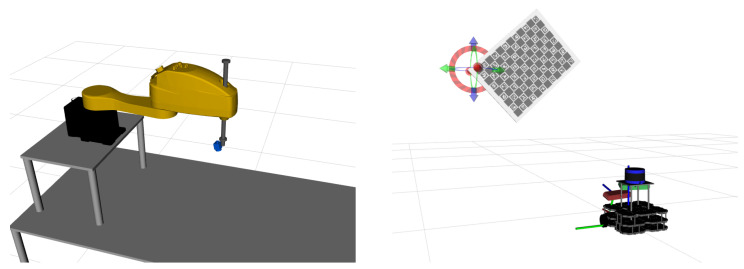
Two examples of systems that have been calibrated by ATOM. On the left, there is spjbot, a system consisting of a robotic manipulator with prismatic joints with an RGB camera mounted on its end-effector. On the right, there is softbot, a robotic vehicle equipped with a 3D Light Detection and Ranging (LiDAR) sensor and two cameras.

The target for testing by the pipeline proposed in this paper is ATOM, as mentioned. ATOM is a calibration software for generalized robotic systems. It is capable of handling several types of calibration: *sensor to sensor*, *sensor in motion* and *sensor to frame* [2]. This system is based on the idea of indivisible transformations. Generally, a solution to a calibration problem involves simplifying the topological tree of transformations, given a specific situation and system. However, ATOM does the opposite, preserving the complexity of a system’s transformation tree to achieve generalization. Examples of systems calibrated using ATOM (https://github.com/lardemua/atom/tree/noetic-devel/atom_examples (accessed on 4 March 2025)) can be found in Figure 1.

The ATOM calibration process comprises multiple steps. First, the calibration is configured via a YAML configuration file. An optional step where an estimate for the sensors’ positioning can be given follows. The next step is not optional, however, and that is the data collection and labeling step. This process outputs a dataset, in the form of a JavaScript Object Notation (JSON) file. This dataset is then used as input to the actual calibration procedure, which outputs another dataset and robot description files, both calibrated. This calibrated dataset is used afterwards in a calibration evaluation process that compares the results to the ground truth and outputs the errors in the calibration.

By creating the proposed pipeline for this software, it is our belief that it would prove to be an effective and useful tool to ATOM’s development team. Furthermore, given the tools and concepts explored thus far, it seems all of the necessary assets for such a pipeline to be created already exist. Most notable of these is Rigel, as its inclusion would simplify the creation of this pipeline considerably.

## 2. Proposed Approach

**Figure 2:**
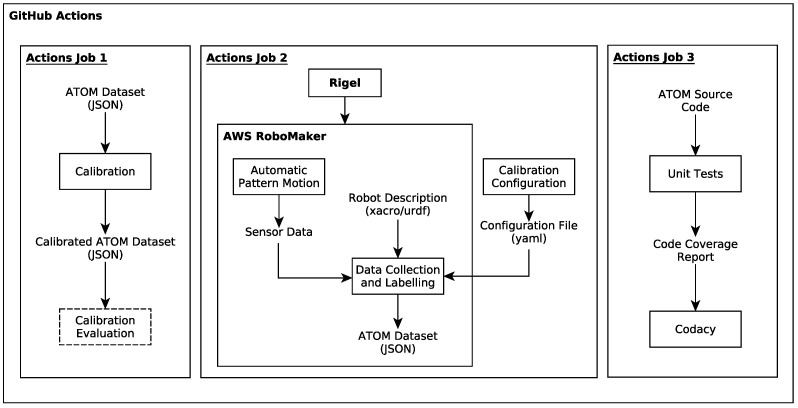
A diagram illustrating how each part of the ATOM calibration procedure will be tested in this pipeline.

As mentioned, the ATOM calibration framework will serve as a testbed for the proposed pipeline in order to validate this approach to applying CI, containerization and cloud simulations to the development of robotic software. The proposed approach to testing ATOM’s calibration procedure is to split the pipeline in two: the data collection and labeling process and the calibration process. A diagram illustrating how each of these parts will be tested can be found in Figure 2.

### 2.1. Robotic System for Testing

ATOM, as mentioned, is an application for the calibration of generalized robotic systems. As such, in order to test its calibration process, a robotic system is needed. Therefore, the first step to creating this pipeline is to choose the robotic systems to test the application. As mentioned, ATOM has been used to calibrate a variety of robotic systems. From among these, three different systems were chosen to be used for the development of this pipeline.

**Figure 3:**
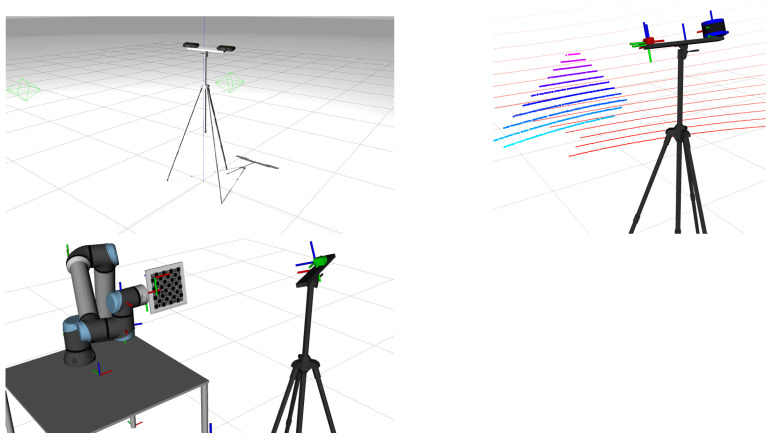
The three robotic systems used to develop the CI pipeline: the t2rgb system (top-left figure), which consists of two RGB cameras in a tripod; the rlbot system (top-right figure), which consists of an RGB camera and a 3D LiDAR in a tripod; the riwbot system (bottom figure), which consists of a robotic manipulator and a tripod with an RGB camera.

The first of these systems is t2rgb, a simple system consisting of a static tripod holding two RGB cameras. The second is rlbot, a system similar to t2rgb, except with a 3D Light Detection and Ranging (LiDAR) instead of a second RGB camera. The third system is riwbot, a system consisting of a robotic manipulator and a tripod with an RGB camera. Figure 3 shows these systems.

### 2.2. Container Image Creation

As illustrated in Figure 2, the data collection and labeling section of the ATOM calibration pipeline will be tested via AWS RoboMaker and the calibration section will be tested via Rigel. For both of these, the containerization of the software, involved in this process as part of the CI pipeline, is necessary.

This means that the next step is to configure the creation of a container image that contains both ATOM and the ROS packages for the necessary robotic systems. This can be completed via the creation of a Rigelfile. As mentioned previously, this is the file that configures the tasks that Rigel executes, named *jobs*. Each job configured in the Rigelfile corresponds to a specific Rigel plugin.

**Figure 4:**
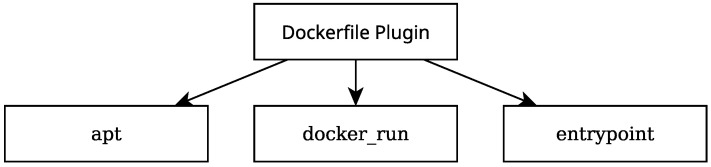
A diagram illustrating the structure of the Rigel *dockerfile* plugin configuration.

To create a container image for testing, two plugins are needed: the *dockerfile* and the *build* plugin. To implement both these plugins, two Rigel jobs need to be configured in the Rigelfile. Figure 4 shows a diagram illustrating the structure of the *dockerfile* plugin section of the Rigelfile. This section of the Rigelfile has several fields. Most notable are the apt, docker_run and entrypoint fields. In the apt field, packages to be installed via the APT package management tool (https://ubuntu.com/server/docs/package-management (accessed on 4 March 2025)) were specified. In the docker_run field, commands are issued to be run when creating the container image. This includes, for example, installing Python packages with pip package installer. The entrypoint field receives a list of commands to be run when the container generated by running the resulting image is orchestrated.

### 2.3. Container Image Orchestration

**Figure 5:**
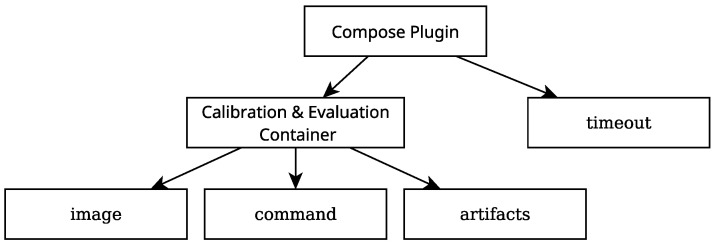
A diagram illustrating the structure of the Rigel *compose* plugin configuration.

After the creation of the container image is configured, the orchestration of said image needs to be configured as well for the testing of the calibration procedure. This is also completed via the Rigelfile by creating a Rigel job that uses the *compose* plugin. This Rigel plugin launches any number of containers. In order to test the ATOM calibration procedure, only one container needs to be launched, from the container image generated by the *dockerfile* and *build* plugins. Figure 5 illustrates the structure of the *compose* plugin section of the Rigelfile. This Rigel job configures a list of containers to launch. For each of these containers, an image is specified and a command is given to be run. A time limit for the testing, after which the container is stopped, is given in the timeout field. The artifacts field receives a list of files to extract from the container during the testing process. In this case, a CSV file is extracted, which contains the results of the calibration evaluation process that will be used later in this pipeline.

### 2.4. The File Introspection Plugin

One of the contributions of this work is a plugin for Rigel, named *file introspection*, designed to inspect and validate values from a file, such as the results file mentioned. It was developed for general use, not just for this specific pipeline. This plugin takes as input (defined in the Rigelfile) a list of column names to analyze through two steps. These are the validation and introspection steps.

When analyzing numerical values in the input CSV file, the validation process compares all of the values in a given column against user-defined thresholds. If the values in the column are not validated, the plugin outputs that information, letting the user know that these results should be discarded and not be taken into consideration. There is no validation process when working with non-numerical values. The introspection process, when working with numerical values, does the same thing as the validation process, except it only validates one of the values in the column in question. For non-numerical values, the plugin checks for the presence of a string in the selected row of the column. Since this plugin is built to be agnostic in regards to its application, it comes down to the user how they wish to use the validation and introspection steps. However, the idea behind the design when dealing with numerical values is the following: the validation process checks if the values are *feasible*, taking into account every value in a column; the introspection process checks if a single value in a column of feasible values is *acceptable*.

**Figure 6:**
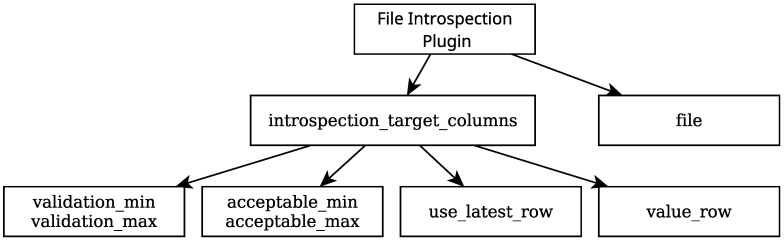
A diagram illustrating the structure of the Rigel *file introspection* plugin configuration.

The structure of the Rigelfile section for this plugin’s implementation can be found in Figure 6. In the file field, the path to the CSV file is given. In this case, it is the calibration evaluation results file, mentioned previously, that is extracted from the container launched by the *compose* plugin as an artifact. Then, a list of columns to be analyzed is given, each with its user-defined parameters. In this case, only the root mean square error column of the evaluation results file is analyzed. The validation process checks if every value in this column is between 0 and 2 pixels. Using either the use_latest_row or value_row parameters allows the user to choose the row of the value to be analyzed by the introspection process. In this case, the average value is selected. Should this value lie between 0 and 3 pixels, the value is deemed acceptable.

### 2.5. Rigel Job Sequence Definition

**Figure 7:**
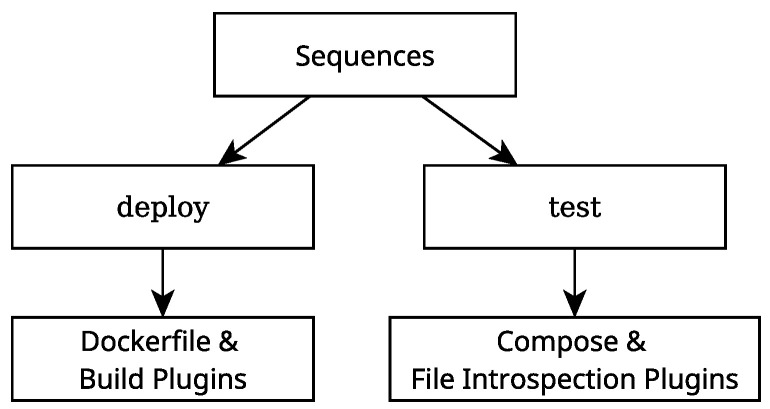
A diagram illustrating the structure of the job sequence section of the Rigelfile.

To use this Rigelfile in this pipeline, the next step is to define several *job sequences*. One of these sequences, named deploy, creates the container image for testing, by using the *dockerfile* and *build* plugins, as defined previously, sequencially. The remaining sequences relate to the testing of the software. When one of these sequences is called, several processes are launched in parallel. Each of these parallel processes call the *compose* and *file introspection* plugins sequentially. Every parallel process runs the calibration process with different values for the initial noise guess parameter. This is carried out so that the testing of the calibration process has increased fidelity. Figure 7 illustrates this. In this case, four parallel sequences are configured to be launched when the test sequence is called, where each of these uses one pair of values for the translation noise and the rotation noise. This pipeline uses the noise values of 0 m and 0.5 m for the translation noise and 0 rad and 0.5 rad for the rotation noise. The calibrations with both initial noise values set to zero serve as a sanity check for ATOM. The calibrations with noise serve to verify that ATOM can respond correctly to noise in its data and provide accurate calibrations despite the noise.

### 2.6. Automatic Data Collection

This section describes our solution to one of the problems faced during the development of this pipeline. On the one hand, CI/CD pipelines operate in a non-interactive manner, meaning the user cannot interact with the process. On the other hand, however, the design philosophy behind ATOM presumes said interaction during certain sections of the ATOM calibration pipeline.

When calibrating a dataset and evaluating the calibration using ATOM, this is not an issue, since no user interaction is required for this process. However, this is not the case when considering the ATOM data collection and labeling process. Thus, in order to include testing for this section of the ATOM pipeline, the option of running this process in an automatic fashion must be made available.

It should be noted that the solution to this problem presented here is specific to the t2rgb system. This is due to the fact that different robotic systems move in different ways and contain different sensor modalities.

To this end, two Python scripts were written. The first script was named motion_recorder. It interactively records poses for the calibration pattern, using RViz. This script was created to ease the use of the automatic data collection for developers and is not used in the CI pipeline. The output of this script is a JSON file that contains the different poses, named the *movement sequence* file. Each pose contains the position of the pattern, as well as its orientation, which is expressed as a quaternion.

The second script is called pattern_motion and it moves the calibration pattern according to the *movement sequence* file. As the script iterates over the *movement sequence* file and moves the pattern, it also automatically saves a collection for the dataset, generated as a result of the data collection and labeling process. It does this by calling ATOM’s ROS service save_collection. By using this script during the data collection process, automation is achieved.

### 2.7. AWS RoboMaker Implementation with Rigel

It is possible to configure the issuing of simulations jobs in RoboMaker with Rigel, by the use of Rigel’s *robomaker* plugin. However, in order to carry this out, some steps need to be taken beforehand.

First of all, two ROS launch files need to be created: one for the *simulation application* and another for the *robot application*. It should be noted that these names sometimes do not reflect what is contained in each application. Typically, the *robot application* contains everything that is the target of testing while the *simulation application* contains everything that supports that testing. In this case, for example, what is being tested is the ATOM data collection and labeling node, so that is what is launched by the *robot application*. In the considered example, the robotic system t2rgb is necessary to run these tests, as well as the automatic data collection node. For this reason, these are included in the *simulation application*.

Secondly, an Amazon Web Service Identity and Access Management (AWS IAM) User and Role must be created for Rigel to access the permissions needed to issue the simulation job. An AWS IAM User represents an individual, or a given automated process that uses AWS’s services. A User can be accessed by using an Access Key. Each Access Key has two components: the Access Key ID and the Secret Access Key (https://docs.aws.amazon.com/IAM/latest/UserGuide/id_users.html (accessed on 4 March 2025)). These two components are provided to Rigel in the Rigelfile’s providers section. An AWS IAM Role, however, is a way to temporarily give an application access to a given service. RoboMaker requires an AWS IAM Role in order to issue a simulation. A Role can be identified with an Amazon Resource Name (ARN), which is an unique identifier which allows for no ambiguity when referencing resources.

**Figure 8:**
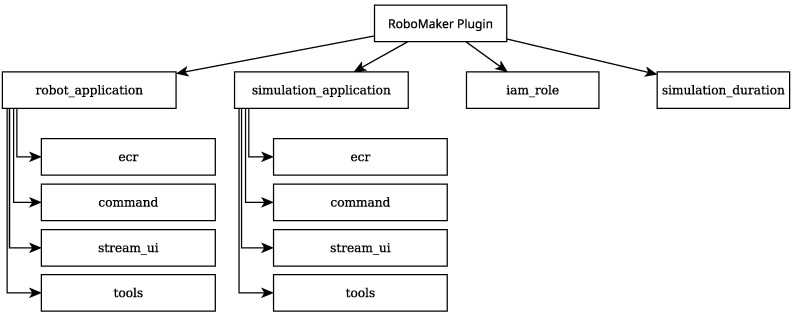
A diagram illustrating the structure of the Rigel *robomaker* plugin configuration.

The structure of the Rigelfile section for this plugin’s integration can be found in Figure 8. Before configuring the two applications, the AWS IAM Role’s ARN is provided, along with a duration for the simulation. For each application, an AWS ECR repository must be provided. The same image is provided for both applications in this case. In the command field, the command to launch the respective application is given. Furthermore, three tools for monitoring are set up: the Gazebo client for the *simulation application* and the terminal and RViz for both.

### 2.8. Unit Testing and Coverage Report

In order to implement unit testing in this pipeline, some unit tests were written for ATOM’s source code. For the purposes of this work, the naming.py module, from the atom_core package, was the target of these tests. Every function in the module was covered, for a total of eight tests. This is carried out to serve as proof of concept for the integration of unit tests in this pipeline. An example of a unit test can be found in Algorithm 1.
**Algorithm 1** Example of a unit test, targeting the function generateName().  A←"prefix"  B←"suffix"  N←"name"  s←"_"**Ensure:** generateName(N,A,B,s)="prefix_name_suffix"

The implementation of these tests in the pipeline is explained in Section 2.9.

### 2.9. CI Workflow Creation and Calibration Testing

**Figure 9:**
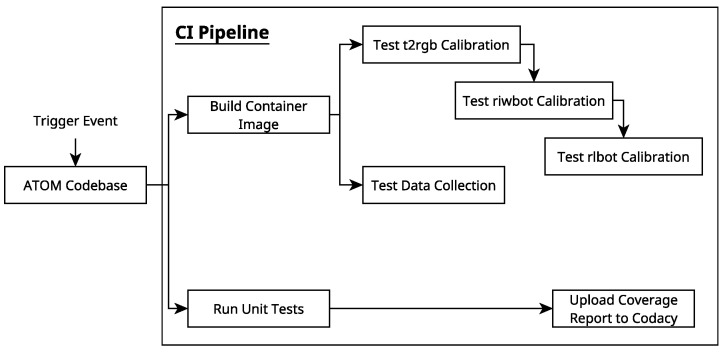
A diagram illustrating the proposed workflow for the CI pipeline.

In order to actually use the Rigelfile written thus far, it is necessary to integrate it in a CI pipeline using GitHub Actions. To achieve this, an appropriate *workflow* must be created. This is carried out via another YAML file. A diagram illustrating the proposed workflow can be found in Figure 9.

This workflow is triggered on a *push* event (i.e., when someone changes something in the repository). Using an ubuntu-latest container, this workflow is composed of three tasks which are called jobs, similarly to Rigel. For the sake of differentiating these jobs from Rigel jobs, they will be referred to as Actions jobs.

The first of these Actions jobs consists of running the calibration procedure on a dataset. To carry this out, the ATOM repository is cloned into the container, and Rigel is installed along with the *file instrospection* plugin. Afterwards, the container runs the calibration via Rigel, inside a container which is itself running inside the GitHub Actions container.

The second of the Actions jobs tests the data collection procedure, by running the Rigel job described in Section 2.7. This job is defined similarly to the first, except for the task run by Rigel.

The third and final Actions job runs the unit tests and creates the code coverage report. It also uploads the created report to Codacy, so as to make the results visible on the platform. To carry this out, the coverage.py module for Python is installed and the unit tests are run using it, which results in a coverage report being generated. To upload the report to Codacy, the Codacy Coverage Reporter GitHub Action (https://github.com/codacy/codacy-coverage-reporter-action (accessed on 4 March 2025)) is used. It should be noted that Codacy needs to be granted access to the source code repository before doing this, which automatically enables the Codacy static analysis features.

## 3. Results

In order to test the process of calibrating a dataset in a CI pipeline, a dataset is needed. Using ATOM, a dataset was manually acquired for the calibration of the t2rgb, rlbot and riwbot systems. These datasets contain several collections, each containing the data from the sensors, as well as the pose of the robot.

By running the calibration and evaluation using Rigel in a GitHub Actions container using our workflow, four parallel instances of the process are run for each robotic system. Each of these instances performs the calibration with different values for the initial noise estimate, for both translation noise and rotation noise. The ATOM calibration evaluation returns the errors in the calibration for each collection in the dataset and their averages. Table 1 shows the average errors acquired for each parallel calibration procedure, as well as the initial noise estimate parameters used.

The errors presented in Table 1 are acquired by computing the root mean square (RMS) reprojection error. Table 1 also contains the results acquired using the OpenCV stereo calibration method (https://docs.opencv.org/3.4/d9/d0c/group__calib3d.html#ga91018d80e2a93ade37539f01e6f07de5 (accessed on 4 March 2025)) for t2rgb, as well as two alternatives to ATOM for the hand–eye calibration of riwbot. These alternatives are the methods presented by Shah [19] and Li et al. [20].

For both t2rgb and riwbot, the calibrations using ATOM achieve lower error values than the other methods presented here. For t2rgb, for instance, ATOM achieves sub-pixel values, while the OpenCV method results in error values above 1.5 pix. Similarly, for riwbot, ATOM achieves errors below 0.75 pix, even for substantial error values. The alternative methods for hand–eye calibration used for this comparison output greater error values.

After the calibration and evaluation process in this Actions job, file introspection is run as part of the same Rigel sequence. In this case, the file introspection plugin is implemented in such a way that only the average RMS values are used. Furthermore, none of these values surpass the maximum threshold values specified for each system in the Rigelfile. As such, the four parallel calibration procedures are validated by the introspection plugin, as expected.

**Figure 10:**
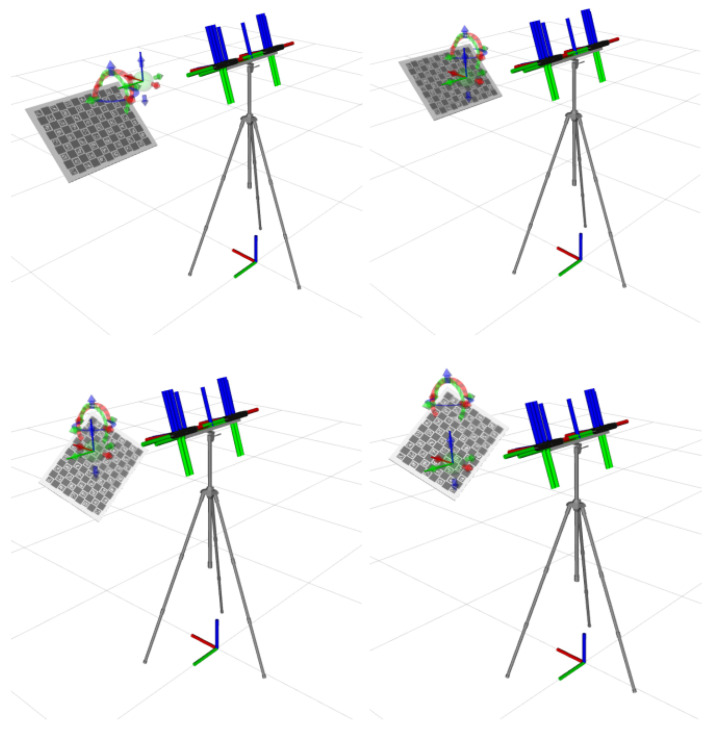
Images captured in RViz, during the process of automatic data collection, using both ATOM and the pattern_motion script. Each image shows the calibration pattern in a different pose during the data collection process.

As previously established, in order to test the data collection process using AWS RoboMaker, the data collection needs to be automatic. Running the automatic data collection process described in Section 2.6 locally allows the capture of images of the pattern in different poses, seen in Figure 10. This behavior is the same when the simulations are run on the cloud, using RoboMaker, as part of the CI pipeline.

Regarding the Codacy static analysis, which is automatically performed, Codacy detected a total of 371 issues in the ATOM source code. Of these issues, 350 were coding style issues, 16 were security-related and for the remaining 5 issues, Codacy pointed to these as prone to error.

**Figure 11:**
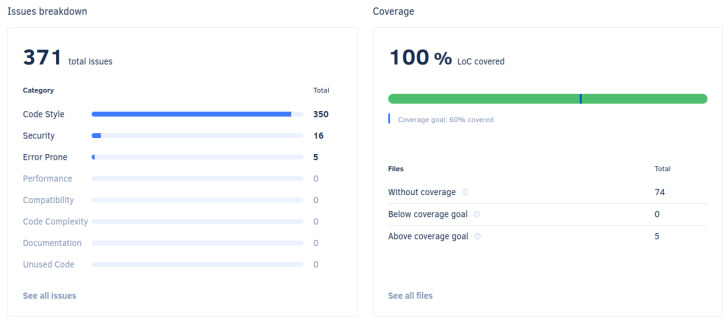
Results from Codacy’s static analysis and the code coverage overview.

In terms of code coverage, 100% of the lines of code of the Python modules which are subject to unit testing are covered. At this point, only the naming.py module is covered. Codacy also points out that 74 source code files do not have coverage, 5 files are above the coverage goal of 60% lines of code covered and also that no files are covered while below the coverage goal. It should be noted that the five files that are above the coverage goal are the following: the naming.py module, the test module that contains the tests for naming.py and three files that were counted as being covered due to not being source code files. These results can be seen in Figure 11.

By collecting the results from the calibration evaluation table and using the file introspection plugin to validate them, it is possible to detect if changes made to the source code of ATOM damage or degrade the calibration process performance in any way. Similarly, the results from the simulations run on AWS RoboMaker show if the data collection and labeling procedure is performed correctly. In these two GitHub Actions jobs, most of the ATOM calibration pipeline is tested. Furthermore, the unit tests run checks to see if the covered modules are working as intended.

Table 2 contains the execution times for each part of the proposed pipeline: the building of the container image; the testing of the calibrations for each of the three systems; the testing of the ATOM data collection process; and the unit tests. Furthermore, the execution times for the complete pipeline are also presented. Table 2 contains data gathered from ten runs of the proposed pipeline, as well as the average values.

As mentioned, our pipeline makes use of an ubuntu-latest container, hosted by GitHub. At the time of writing, these runners for public repositories have 16 GB of RAM and 14 GB of SSD storage. For public repositories, the use of these runners is free. For private repositories, however, there is a cost of USD 0.008 per minute. It is possible to estimate the cost of using this pipeline by multiplying this cost by the mean execution time for the complete CI pipeline. This results in an estimate of USD 0.284 per run.

## 4. Discussion

The purpose of this work was to propose a CI pipeline, used to validate and facilitate the development of robotic applications, and to apply it to the calibration software ATOM. The implementation of such a tool would increase the quality and speed of development, since it automatically tests the functionality of the software.

In this matter, it is the authors’ conclusion that the work was a success. The pipeline that was created is triggered whenever changes are made to the ATOM repository. Three jobs are run when this happens. In one of these, Rigel is used to test ATOM’s calibration procedure, by running several parallel calibrations with different parameters. The second job uses Rigel to test another part of ATOM’s calibration pipeline: the data collection and labeling procedure. The third of these jobs runs all of the unit tests written for ATOM. In this way, most of ATOM calibration pipeline is addressed by this testing pipeline.

Furthermore, Codacy is used to perform static analysis on ATOM’s repository. It also displays the results from the unit testing job through a unit test coverage report.

As a result of this work, some improvements were made to Rigel. Firstly, the *file introspection* plugin is a direct contribution of this work to Rigel’s body of plugins. Secondly, indirect contributions were made to Rigel’s development by inspiring some new features that are present in this work. These include the possibility of running parallel jobs and the extraction of files from containers as artifacts in the *compose* plugin.

However, some work can still be carried out, since there are clear avenues for improvement. The first of these would be to include a CD mechanism, so that ATOM is automatically deployed to a container registry or simply as an automatically versioned release on GitHub.

Furthermore, there is a part of ATOM’s pipeline that is not tested, which is the setting of an initial estimate for the sensors. Including the testing of this part of the calibration procedure in the pipeline would be one of the next steps in the development of this tool.

On the topic of unit testing, tests were written for one of ATOM’s Python modules, as a proof of concept. One of the more immediate improvements that could be made is to include more unit tests in ATOM. Ideally, every Python module would be covered by unit tests.

The biggest improvement that could be made, however, is to take advantage of the parallelism potential of running simulations on the Cloud. Presently, only one simulation is run using AWS RoboMaker at a time. However, several could be run in parallel, testing in different environments. In this specific case, that might mean the calibration pattern having different motion sequences for each of the data collection simulations. However, it is the authors’ belief that this boon of Cloud simulations can be used to its full effect by using a tool like AWS RoboMaker WorldForge (https://aws.amazon.com/blogs/aws/aws-announces-worldforge-in-aws-robomaker/ (accessed on 4 March 2025)). This is a tool for creating different simulation worlds, with a degree of randomization. By using both WorldForge and Rigel’s capability for parallelism, this CI pipeline could benefit from issuing several simulations on the Cloud with different environments.

Overall, it is the authors’ belief that this pipeline is a valuable tool for the development of ATOM. The increase in software quality that this pipeline facilitates, through consistent and systematic testing, is expected to have a positive impact in the overall quality of calibrations undertaken using ATOM, by helping developers to avoid potential bugs or other failings which might result in the degradation of the calibration results. The pipeline can also serve as a basis for similar pipelines for the development of other ROS applications, being able to cover a wide variety and volume of tests by making use of tools like Rigel and AWS RoboMaker.

## 5. Conclusions

In this work, we present a framework for the development of ROS applications, based on the concept of Continuous Integration. We developed a CI pipeline for the testing of an application for the calibration of generalized robotic systems, ATOM. Our pipeline integrates Rigel to simplify testing and enable parallel testing through the simultaneous orchestration of several containers. It also integrates AWS RoboMaker, which allows testing with Gazebo simulation environments in the Cloud. Static code analysis and unit testing are also implemented in our pipeline, for a more complete testing procedure. Nonetheless, this pipeline can be improved upon in future works, and the potential boons of running simulations in the cloud can be further explored. Different directions for potential improvement were given in the previous section.

We believe that this pipeline serves as a powerful tool for the developers of ATOM, by testing the majority of the ATOM calibration pipeline whenever a change is made to the source code repository. It is also our belief that this pipeline will prove to be a good basis for the developers of other ROS applications to create their own pipelines.

## Figures and Tables

**Table 1:** The average errors of four parallel calibrations run on a dataset, and the corresponding values for their initial noise estimates. These calibrations are run simultaneously as a part of the CI pipeline. In addition to these four calibrations, the results for the OpenCV stereo calibration are presented for t2rgb, as well as the results from two alternative calibration methods for riwbot.

Method	Trans. Noise	Rot. Noise	RMS Error (pix)		
			t2rgb	riwbot	rlbot
ATOM	0.0	0.0	0.6000	0.7161	2.8133
	0.5	0.0	0.6009	0.7418	2.8094
	0.0	0.5	0.6004	0.7205	3.1954
	0.5	0.5	0.6007	0.7315	2.8443
OpenCV	0	0	1.6510	—	—
Shah [19]	0	0	—	0.8317	—
Li et al. [20]	0	0	—	1.3206	—

**Table 2:** Execution times for each part of the proposed pipeline. Presented here are the execution times gathered from ten runs of the proposed pipeline, as well as their average values.

Run #	Build	Calibration			Data Collection	Unit Test	Complete
		t2rgb	rlbot	riwbot			
#1	17 m 32 s	7 m 43 s	5 m 5 s	5 m 4 s	7 m 43 s	16 s	36 m 47 s
#2	16 m 30 s	7 m 7 s	5 m 5 s	5 m 5 s	7 m 50 s	15 s	35 m 13 s
#3	17 m 3 s	7 m 3 s	5 m 5 s	5 m 4 s	7 m 53 s	31 s	35 m 46 s
#4	19 m 19 s	6 m 48 s	5 m 5 s	5 m 4 s	7 m 51 s	19 s	37 m 50 s
#5	16 m 0 s	6 m 46 s	5 m 5 s	5 m 5 s	8 m 4 s	16 s	34 m 21 s
#6	17 m 23 s	6 m 46 s	5 m 4 s	5 m 5 s	7 m 55 s	15 s	35 m 47 s
#7	17 m 47 s	6 m 46 s	5 m 4 s	5 m 4 s	7 m 59 s	15 s	36 m 14 s
#8	15 m 53 s	6 m 54 s	5 m 4 s	5 m 4 s	7 m 47 s	21 s	34 m 13 s
#9	16 m 52 s	6 m 44 s	5 m 5 s	5 m 5 s	7 m 39 s	14 s	35 m 1 s
#10	16 m 11 s	6 m 51 s	5 m 5 s	5 m 4 s	7 m 53 s	14 s	34 m 25 s
**Average**	17 m 3 s	6 m 57 s	5 m 5 s	5 m 4 s	7 m 51 s	18 s	35 m 34 s

## Data Availability

Data are contained within the article.
